# Case Report: Systemic Amyloidosis Involving the Heart and Skeletal Muscle

**DOI:** 10.3389/fcvm.2022.816236

**Published:** 2022-04-04

**Authors:** Pinchao Lv, Yuxi Li, Lin Wu, Qiuping Shi, Lingchao Meng, Xiaojuan Yu, Lin Nong, Jianping Li

**Affiliations:** ^1^Department of Cardiology, Peking University First Hospital, Beijing, China; ^2^Department of Neurology, Peking University First Hospital, Beijing, China; ^3^Department of Nephrology, Peking University First Hospital, Beijing, China; ^4^Department of Pathology, Peking University First Hospital, Beijing, China

**Keywords:** amyloidosis, transthyretin, myopathy, biopsy, heart failure, light chain

## Abstract

**Background:**

Amyloidosis refers to an etiologically heterogeneous group of protein misfolding diseases characterized by extracellular deposition in organs and tissues of amyloid fibers, leading to severe organ dysfunction and death. Systemic amyloidosis often involves multiple organs. Heart and kidney are the most commonly affected organs, whereas skeletal muscle involvement is rare and often accompanied by other organs’ involvement.

**Case Summary:**

We reported a 70-year-old man manifested with myopathy followed by heart failure who was suspected of transthyretin amyloidosis clinically, after the pathological results and the ^99m^Tc-pyrophosphate (^99m^Tc-PYP) scintigraphy, light-chain (AL) amyloidosis involving the heart and skeletal muscle was confirmed.

**Conclusion:**

The patient’s unique presentation gives insight into a rare but debilitating disorder and the potential link between various types of amyloidosis. In addition, myopathy in amyloidosis should be recognized.

## Introduction

Amyloidosis is mainly divided into monoclonal immunoglobulin light chains (AL), transthyretin (TTR) and secondary to chronic inflammatory and infectious diseases (AA), etc. according to the chemical characteristics of the precursor protein (1). Cardiac amyloidosis (CA) is most common in AL/systemic and ATTR amyloidosis, which is a severe progressive disease caused by the deposition of amyloid fibrils in the heart, often accompanied by other extracardiac manifestations (2). However, skeletal muscle involvement alone is rare in systemic amyloidosis and often coexists with peripheral neuropathy. Thus, the classification of amyloidosis involving the skeletal muscle without peripheral neuropathy is challenging in clinical practices. Different types of amyloidosis have distinct treatments. The treatment of AL amyloidosis is mainly chemotherapy aimed at the plasma cell dyscrasia, which can delay the disease progression and prolong the survival time, and the prognosis remains poor (3). As for ATTR amyloidosis, effective therapies inhibit TTR synthesis and stabilize TTR molecule, among which tafamidis is one of the selective TTR stabilizers that improves outcomes in patients with ATTR-CA (4).

## Case Presentation

**Table:** Timeline of the patient’s clinical course

Initial presentation	• Backache after activity
1 year	• elevation of CK was noted, 921.8 IU/L (normal range 25–170 IU/L)
1.5 year	• CK-MB 22.7 ng/ml (normal range <5 ng/ml) • cTNI 0.047 ng/ml (normal range <0.03 ng/ml) • ECG first-degree atrioventricular block • MRI (thigh) showed fat infiltration and mild edema in bilateral hip and thigh muscles • Coronary angiography revealed triple vessel disease and one stent was implanted in LCX
2 year	• Persistent elevation of CK • Myopathy of unknown etiology was diagnosed
2.5 year	• Exertional dyspnea, bilateral lower extremity edema • TTE showed symmetrical left ventricular hypertrophy, LVEF 58.7% • Cardiac MRI considered subacute myocarditis

*CK, creatine kinase; CK-MB, creatine kinase isoenzyme; cTNI, cardiac troponin I; ECG, electrocardiogram; MRI, magnetic resonance imaging; LCX, left circumflex artery; TTE, transthoracic echocardiography; LVEF, left ventricular ejection fraction.*

**FIGURE 1:**
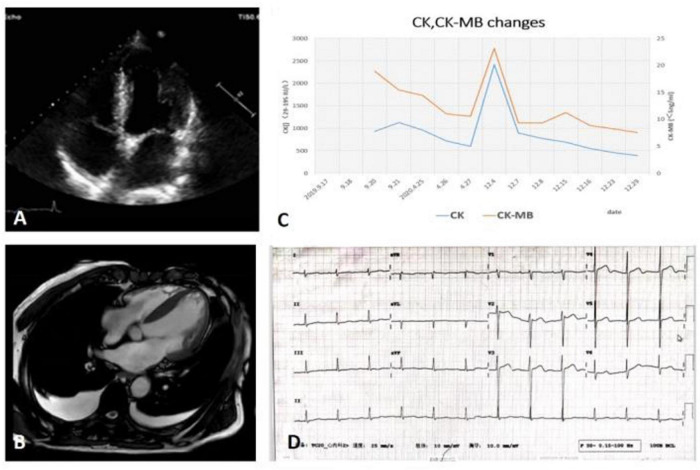
**(A)** TTE: Left and right ventricular wall thickening with normal LVEF, diminished left ventricular diastolic function, and right ventricular systolic function. **(B)** CMR: Multiple sites of myocardial edema and delayed enhancement. **(C)** The trend of CK, CK-MB of patient. **(D)** The ECG on admission. CK, creatine kinase; CK-MB, creatine kinase isoenzyme; CMR, cardiac magnetic resonance; ECG, transthoracic echocardiography; LVEF, left ventricular ejection fraction; TTE, transthoracic echocardiography.

A 70-year-old male was admitted to the hospital in December 2020 due to “dyspnea for 10 days.” The patient had exertional dyspnea 10 days ago, which was progressively worsening with paroxysmal nocturnal dyspnea, and bilateral lower extremity edema. Cardiac MRI showed myocardium with edema in the inferior wall of basal left ventricular, middle and anterior of apex, together with multiple patchy delayed enhancement areas in the anterior wall of basal left ventricular, septal and inferior wall, leading to suspicion of non-ischemic cardiomyopathy, and subacute myocarditis (as shown in Figure 1). The patient’s weight gained nearly 7 kg in a month. He had a history of diabetes and smoking, but no family history of neuromuscular or heart disease. As shown in the timeline, the patient initially presented with back pain and was found with an elevation of creatine kinase (CK) without any myalgia or muscle weakness 1 year after the initial presentation; meanwhile, thyroid function test, anti-thyroid antibodies, and myositis antibody spectrum were normal, and no statin medication history. MRI of the thigh showed MR changes in bilateral hip and thigh muscles, with fat infiltration and mild edema. The patient was performed coronary angiography, which showed triple-vessel disease (distal left anterior descending artery 80% stenosis, distal right coronary artery 60–70% stenosis, and distal left circumflex artery 80% stenosis), and implanted one stent in the left circumflex artery (LCX). The patient’s back pain was slightly relieved after interventional therapy, but the CK continuously increased (as shown in Figure 1), creatine kinase isoenzyme (CK-MB) and cardiac troponin I (cTNI) fluctuated at a low level, and cardiac troponin T (cTNT) values were normal. A neurologist suspected myopathy and suggested an electromyography and muscle biopsy to confirm the diagnosis. The autoantibody and myositis antibody spectrum showed ANA 1:100 (H + S), anti-Scl-70 antibody (+), and anti-JO-1 antibody (+).

### Admission Physical Examination

Stable vitals, abdomen skin was thickened and wrinkled, stale rash on the back, bilateral loins and tibia of both lower limb, rough breathing sounds in lungs and low breath sound in the right lung, normal regular heart rhythm and normal heart sounds, bilateral lower extremity edema, interphalangeal joint of right ring finger tenderness and limited movement, and tight and stiff skin of both hands. No mask face or mouth rays.

### Laboratory Tests

Normal blood routine examination, abnormal liver function (alanine transaminase 53 IU/L, glutamic oxaloacetic transaminase 1,02 IU/L, and gamma-glutamyl transpeptidase 94 IU/L), normal renal function for his age (creatinine 95.30 μmol/L, estimated glomerular filtration rate 69.324 ml/min/1.73 m^2^), and elevated cardiac biomarkers (CK-MB 23.1 ng/ml, cTNI 0.089 ng/ml, and BNP 679.00 pg/ml). CK 2,413 IU/L, lactic dehydrogenase 430 IU/L, high sensitivity CRP 4.22 mg/L; no proteinuria; urine α1-microglobulin 16.90 mg/L; 24 h urine protein: 0.04 g/24 h (1,100 ml); immunoglobulin G (IgG) 6.25 g/L, IgM 0.17 g/L; protein electrophoresis of serum and urine (−); serum immunofixation electrophoresis (IFE) (−), urine IFE: monoclonal light chains IgG κ (+); serum total light chain: κ 502.00 mg/dl (598–1,329 mg/dl), λ 474.00 mg/dl (280–665 mg/dl), κ/λ = 1.06; autoantibody and myositis antibody spectrum: ANA 1:100 (H + S), anti-Scl-70 antibody (+), the others were negative; ECG: first-degree AVB, PR interval 245 ms; TTE: symmetrical left ventricular hypertrophy (ventricular septum 15 mm, left ventricular posterior wall 13 mm, and right ventricular free wall 6 mm), left ventricular ejection fraction (LVEF) 58.7%, E/A 2.2, and E/E′ 21.1. The hand X-ray showed degeneration; the ventilation-perfusion scan showed no pulmonary embolism; ^99m^Tc-PYP (^99m^Tc-PYP) scintigraphy was negative; and TTR gene sequencing of the blood did not identify any mutation.

### Myocardial Biopsy

**FIGURE 2:**
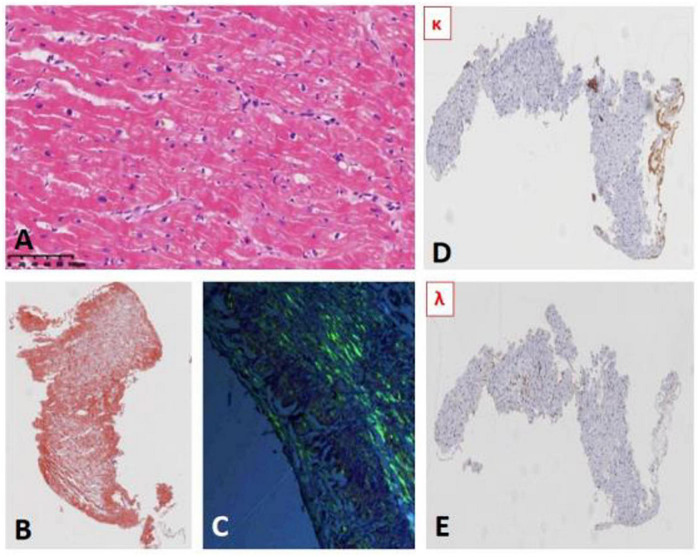
**(A)** Myocardial hematoxylin and eosin (HE) staining. **(B)** Myocardial Congo red staining (HE): Deposition of brick red material. **(C)** Myocardial Congo red staining (polariscope): apple-green birefringence. **(D,E)** Myocardial immunohistochemical results: light κ positive **(D)** Light λ negative **(E)**.

Cardiac fiber hypertrophy, deposition of lipofuscin in cytoplasm, perinuclear hyalinosis, and contraction band necrosis were observed; and amyloid deposition (Congo red staining showed the characteristic apple-green birefringence under polarized light) with positive light chain κ in subendocardium. No signs of myocarditis were observed (as shown in Figure 2).

### Skeletal Muscle Biopsy (Left Quadriceps)

**FIGURE 3:**
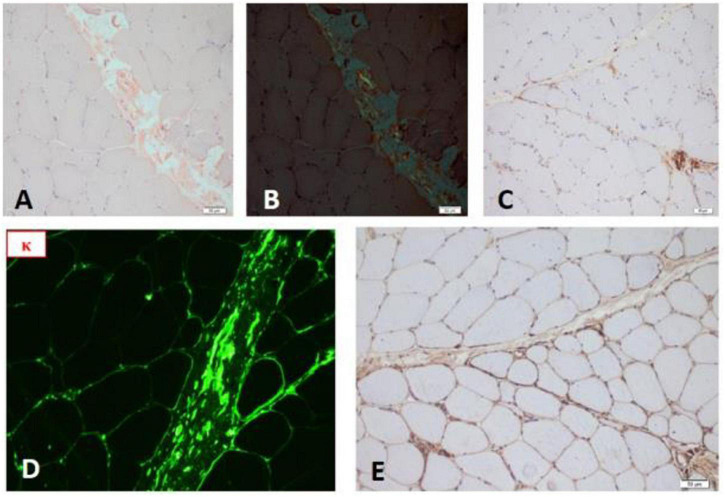
**(A)** Congo red staining of skeletal muscle. **(B)** Congo red staining (polariscope). **(C)** Complement deposition. **(D)** Frozen fluorescence showed skeletal muscle stained strongly for κ light chain. **(E)** TTR gene staining showed negative.

Mycopathological changes, a few atrophy, and regenerated muscle fibers were observed. There was a few positive expression of MHC-1 in muscle fibers and the deposition of membranous complement in non-necrotic muscle fibers in the perifascicular area, which was consistent with the characteristics of myopathy pathological changes. A small number of cytochrome oxidase (COX)-negative muscle fibers were observed, considered as aging changes. No pathological changes of typical muscular dystrophy and neurogenic skeletal muscle were found (as shown in Figure 3).

### Bone Marrow Biopsy

**FIGURE 4:**
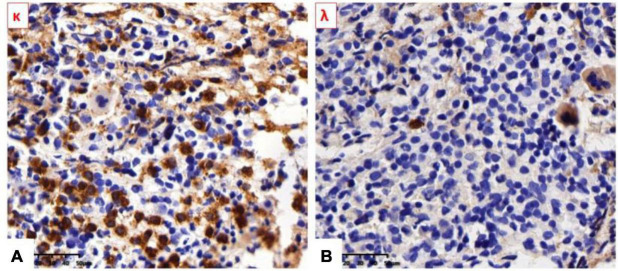
**(A)** κ Light-chain stain was strongly positive. **(B)** λ Light-chain stain was negative.

No obvious morphological abnormality was observed in all lines of cells. More plasma cells were scattered or infiltrated in the interstitium (CD138^++^, κ^++^, λ^–^, VS38c^++^, and MUC1^–^) and accounting for about 25%. Hyperplasia of myeloid plasma cells with light chain restriction expression and mild myelofibrosis, which considered to be a plasma cell disorder (as shown in Figure 4).

### Diagnosis and Therapy

Cardiac amyloidosis and myopathy due to primary AL amyloidosis. Chemotherapy with bortezomib + cyclophosphamide + dexamethasone (BCD) regimen was then initiated in other hospitals.

## Discussion

In our case, the patient was an elderly male over 70 years old, presenting with persistent elevated CK at the beginning, then heart failure with preserved ejection fraction (HFpEF) and autonomic manifestations (postural hypotension). An abnormal IFE κ/λ ratio in serum was found. Amyloid deposits in myocardium and skeletal muscle without evidence of renal involvement. It is well known that CA is most common in AL amyloidosis and ATTR amyloidosis especially wild-type (ATTRwt) (5, 6). The extracardiac manifestations of amyloidosis vary according to the types of amyloidosis, and the amyloid nephropathy is more common in AA and AL amyloidosis, but rare in ATTR amyloidosis (7). Patients with ATTRwt are older than patients with AL amyloidosis (as shown in Supplementary Material). Thus, it seems the clinical manifestations of the patient tend to be ATTR amyloidosis.

However, the pathological results of myocardial, skeletal muscle, and bone marrow showed deposition of light chain κ, which was not in accord with our expectations. Yet, it has been reported that the presence of coexistence of AL amyloidosis and ATTRwt in the literature (8), even the concurrence of ATTR and monoclonal gammopathy (MG). In other words, an abnormal IFE κ/λ ratio in older men may cause diagnostic confusion in amyloid types (9). Therefore, to ascertain whether ATTR coexists, we recommended scintigraphy, TTR gene sequencing, and stained the skeletal muscle for TTR, and the results were negative (as shown in Figure 3). So far, we excluded the ATTR and confirmed the diagnosis of light chain amyloidosis. In general, the patients with ATTRwt-CA have a slower progression, which takes 5–10 years. Instead, the progression of AL-CA is relatively fast. The rapid progression of heart failure in our patient (6 months) reinforced our diagnosis.

Another aspect worth discussing is the amyloid myopathy. In our case, the presence of clinical heart failure was preceded by a sustained elevation of CK, lacking muscle weakness, or myalgia, and MRI did not show the typical reticular subcutaneous fat of amyloid myopathy (10, 11), but we found the muscle complement deposition and Congo red staining positive results (Figure 3). Actually, amyloid myopathy is rarely involved in systemic amyloidosis (12). They often coexist with peripheral neuropathy, and the latter is more commonly involved than skeletal muscle. Among which the AL and ATTRv are the most common types of amyloidosis that affect skeletal muscle (6). Even so, amyloid myopathy was reported in only 1.5% of patients with AL amyloidosis, which may be related to the fact that Congo red staining was not part of our standard muscle biopsy panel on muscle biopsies (13). It has been found that routine Congo red staining in all diagnostic muscle biopsies is 10 times more likely to diagnose amyloid myopathy than selective Congo red staining in clinically or pathologically suspicious cases (14). The typical amyloid myopathy has various manifestations rather than no symptoms like our patient. In the Mayo Clinic, a 20-year study of patients with systemic amyloid myopathy summarized the common clinical manifestations of amyloid myopathy: proximal muscle weakness, myalgia, amyotrophy, pseudo-muscular hypertrophy, muscle claudication, tenderness, muscle contracture, activity decreased, weight loss, fatigue, dysphagia, dyspnea, macroglossia, etc. (15). Although most myopathies have elevated CK, which is not a reliable indicator in the diagnosis of amyloid myopathy because about two-thirds of patients with systemic amyloid myopathy have normal CK levels (10). However, the CK in our case was high and exceeded the median value (as shown in Supplementary Material). Furthermore, amyloid myopathy can be an initial manifestation of ATTRwt (6). And our patient presented with only the elevated CK as the initial manifestation of AL amyloidosis (16). Reviewing the clinical course of the patient, if a muscle biopsy and Congo red staining was performed at the beginning of CK elevation, the patient may be treated earlier. The classification of amyloidosis and the respective characteristic of amyloid myopathy are summarized in Supplementary Table 1.

It is also notable in this case that the complex relationship between connective tissue disease and amyloidosis should be concerned given the multiple systems involvement in this patient: cardiac (HFpEF, first-degree AVB), respiratory (type I respiratory failure), serous cavity (pleural effusion), skeletal muscular (elevated CK), and skin (thin, tight, and limited movement), accompanied by a transient increase of ANA +, anti-Scl-70, and anti JO-1 antibody. In the early period, we also suspected of autoimmune system diseases, such as scleroderma, polymyositis, dermatomyositis, inclusion body myositis, and rheumatoid arthritis. Many types of amyloidosis are known to be associated with muscular-articular diseases, including AL, TTR, and β-2 microglobulin amyloidosis (17). In rare cases, patients may exhibit clinical features similar to those of known rheumatic diseases, which lead to misdiagnosis. Cases of amyloidosis misdiagnosed as polymyositis and scleroderma have been reported in the literature (15, 18). And amyloidosis also can be coexisted with or secondary to connective tissue disorders (19–21). The musculoskeletal manifestations caused by amyloid deposition may be subtle and subclinical, which may be detected only by biopsy. The case was evaluated by a rheumatologist consultation, considering there were no specific scleroderma characteristics currently, the diagnosis of scleroderma is not supported temporarily. The anti-Scl-70 may be false positive as which in multiple myeloma patients with cryoprecipitate (22). Even so, the patient needs to be followed up for a long time to prevent concurrence of connective tissue disease.

## Conclusion

We reported a senior man who was suspected of ATTR amyloidosis clinically, but the pathological results confirmed the diagnosis of AL amyloidosis involving the heart and skeletal muscle. Cardiac amyloidosis is one of the most commonly affected organs in systemic amyloidosis, while amyloid myopathy is relatively rare. This case was meaningful in that it highlights the importance of fully recognizing disparate clinical characteristics of various types of amyloidosis and improving awareness of amyloid myopathy in practice.

## Data Availability Statement

The original contributions presented in the study are included in the article/Supplementary Material, further inquiries can be directed to the corresponding author/s.

## Ethics Statement

The studies involving human participants were reviewed and approved by the Biomedical Research Ethics Committee, Peking University First Hospital. The patients/participants provided their written informed consent to participate in this study.

## Author Contributions

PL was writing the main part of this manuscript. YL had an equal contribution to this case report. JL was supervising PL and YL. LW was investigating the novelty and the rarity of this case. QS was responsible for clinical diagnosis and treatment of patient. LM, XY, and LN were providing the relevant pathological data. This case report article was carried out in collaboration between all coauthors. All authors read and approved the final manuscript.

## Conflict of Interest

The authors declare that the research was conducted in the absence of any commercial or financial relationships that could be construed as a potential conflict of interest.

## Publisher’s Note

All claims expressed in this article are solely those of the authors and do not necessarily represent those of their affiliated organizations, or those of the publisher, the editors and the reviewers. Any product that may be evaluated in this article, or claim that may be made by its manufacturer, is not guaranteed or endorsed by the publisher.
