# Supplementary material for: Case Report: Systemic Amyloidosis Involving the Heart and Skeletal Muscle
Source: Front Cardiovasc Med. 2022 Apr 4;9:816236. doi: 10.3389/fcvm.2022.816236 (PMC9013752; doi:10.3389/fcvm.2022.816236)
Supplement: Supplementary file 1 [file Table_1.docx]

Supplementary materials

Table 1.summary of main features of AL vs ATTR and the patient (6, 23)

| type | AL | ATTR | The patient |
| --- | --- | --- | --- |
| Precursor protein | Immunoglobulin light chain | Transthyretin | / |
| age | median age 63 (slight male predominance) | ATTRwt: over 75  (male predominance) ATTRv: variable (equal to slight male predominance) | **70** |
| monoclonal gamma-globulinemia | (+) | (-) | (+) |
| bisphosphonate scintigraphy | (-) | (+) | (-) |
| TTR mutation | (-) | ATTRwt (-) ATTRv (+) | (-) |
| cardiac amyloidosis |  |  | (+) |
| frequency | 70% | 100% in ATTRwt 30%-100% in ATTRv | / |
|  |  |  |  |
| progression | fastest | relatively slow | 6 month |
| amyloid myopathy |  |  | (+) |
| frequency | 1.50% | very rare | / |
| symptoms | Proximal and distal muscle weakness, dysphagia | Proximal, distal and axial muscle weakness | **no obvious symptoms** |
| coexistence of peripheral neuropathy | 40% | 90% | **(-)** |
| median CK  (range),x ULN | 0.77 | 0.4 | **3.9** |
| renal involvement | Common (50-70%) | rare | **(-)** |
